# Watch Me Grow: A garden-based pilot intervention to increase vegetable and fruit intake in preschoolers

**DOI:** 10.1186/1471-2458-13-363

**Published:** 2013-04-18

**Authors:** Rebecca J Namenek Brouwer, Sara E Benjamin Neelon

**Affiliations:** 1 Duke Global Health Institute, Duke University, 310 Trent Hall, Durham, NC 27710, USA; 2 Department of Community and Family Medicine, Duke University Medical Center, 2200 W Main St, DUMC 104006, Durham, NC 27705, USA

## Abstract

**Background:**

Americans, including children, consume fewer fruit and vegetable servings than is recommended. Given that young children spend large amounts of time in child care centers, this may be an ideal venue for increasing consumption of and enthusiasm for fruits and vegetables. This pilot study aimed to assess the feasibility of a gardening intervention to promote vegetable and fruit intake among preschoolers.

**Methods:**

We enrolled two intervention centers and two control centers. The intervention included a fruit and vegetable garden, monthly curriculum, gardening support, and technical assistance. We measured mean (SD) servings of fruits and vegetables served to and consumed by three children per center before and after the intervention.

**Results:**

Post intervention, intervention and control centers served fewer vegetables (mean (standard deviation) difference of -0.18 (0.63) in intervention, -0.37 (0.36) in control), but intervention children consumed more than control children (+0.25 (1.11) vs. -0.18 (0.52). The number of fruits served decreased in all centers (intervention -0.62 (0.58) vs. control -0.10 (0.52)) but consumption was higher in controls (intervention -0.32 (0.58) vs. control 0.15 (0.26)).

**Conclusions:**

The garden-based feasibility study shows promise, but additional testing is needed to assess its ability to increase vegetable and fruit intake in children.

## Background

Fruit and vegetable intake is a key factor in preventing major illnesses such as cardiovascular diseases and certain cancers, yet the majority of Americans, including children, consume far less than the recommended number of servings per day [1,2]. Researchers have found that certain variables drive fruit and vegetable preference beginning in early childhood, and hence consumption, in young children. In infancy, children show preferential interest in sweet foods, such as sugar solutions and fruit, relative to foods with more bitter flavors [3-5]. Consumption of more bitter vegetables, such as dark leafy greens, is likely driven by repeated exposures, positive role modeling, and learned experiences. Forestell *et al*[6] found that maternal consumption of vegetables during breastfeeding and subsequent weaning influenced children’s later acceptance of a non-sweet food (green beans). Additional evidence suggests that offering a range of healthy foods to young children is likely to result in their increased willingness to taste, and ultimately consume new foods such as certain vegetables [7-13]. Role modeling by adults and children can also have a positive effect on consumption of healthy foods, not merely by eating such foods in front of children [8], but also by reinforcing the experience with positive language [14].

Exposing children to a variety of fruits and vegetables in early childhood and engaging them in the process of growing their own produce may increase habitual consumption throughout life [15,16]. There is evidence that preschool-age children eat more fruits and vegetables when they have access to gardens [17,18]. A recent review by Robinson-Obrien *et al*[19] suggests that garden-based programs are a promising avenue for increasing fruit and vegetable consumption for school-age children. However, less is known about this approach for younger children and few interventions have targeted child-care environments, where large numbers of children spend time.

Nearly two thirds of preschool-aged children are routinely cared for outside of the home [20], suggesting that organized child care is likely a key setting for exposing children to healthy foods. Intervention is important given that fruit and vegetable consumption is low in child care. A recent study [21] found that children consumed only one third of a serving of fruit and one quarter of a serving of vegetables per day while in child care; these findings are consistent with other studies examining fruit and vegetable intake in child care [22,23].

The purpose of this study was to assess the feasibility of a garden-based intervention to promote fruit and vegetable intake among children three to five years of age attending child care.

## Methods

### Study design and participants

We conducted a randomized, controlled trial for this four-month-long pilot study. Participants included center directors and children from four licensed child-care centers located in central North Carolina. To participate in the study, centers had to provide all foods and beverages to children in care (i.e., parents could not send food from home), not have an open case of abuse or neglect with the state licensing agency, and have at least three children between the ages of three and five years in care on a regular basis. We mailed a letter of invitation to every licensed center (n = 6) in the city limits of a small community near our research offices. The letter was followed by a telephone call from the study team. We enrolled the first four centers that agreed to participate. Center directors provided written informed consent to participate in the study; parents were provided a fact sheet describing the study and were asked to contact the project director if they did not want their children observed during the dietary assessment. The Institutional Review Board of the Duke University Medical Center approved this study.

Prior to randomization, we conducted the dietary assessments over two days at each center, targeting three children from one classroom per center for observation. We did not collect any identifying information on the children except age in years and child sex. After baseline data collection, we randomized centers via a simple randomization scheme (randomization without restriction) to either the intervention or control condition on a 1:1 ratio, using the Research Randomizer (http://www.randomizer.org/form.htm). Dietary assessments were conducted again approximately five months after baseline, to coincide with the end of intervention activities. Control centers received a delayed intervention, excluding technical assistance visits, after the final dietary assessment.

### Intervention

The Watch Me Grow program is a garden-based intervention aimed to increase the number of vegetables and fruits provided to and consumed by children in child care. The intervention took place in spring 2011. The program includes a “crop-a-month” structured curriculum for child-care providers, consultation by a gardener, and technical assistance from a health educator. Over the course of the four-month-long intervention, providers and children in the intervention centers grew (1) lettuce, (2) strawberries, (3) spinach, and (4) broccoli. We designed the garden to yield one crop per month, and provided classrooms in the intervention centers with corresponding curriculum materials highlighting the target fruit or vegetable of the month.

Prior to installation, we conducted site visits to determine placement of the garden. Appropriate locations included adequate sunlight (i.e., at least eight hours per day), a nearby water source (e.g., near a hose or a door to the kitchen), and protection from wildlife. We planned for containers and raised beds with soil provided by the study team due to safety concerns about potential contaminants. We computed the square footage needed to grow each crop and yield at least one serving of the target fruit and vegetable per child in the center. The study provided all garden supplies, including plants, watering cans, brackets, and materials for the raised bed. We installed an 8′ × 4′ raised bed outdoor garden at each center. We selected our four target crops based on skill needed to grow them (i.e., minimal), child acceptance and palatability, and appropriateness to the zone seven growing climate in North Carolina. We opted not to plant certain fruits and vegetables due to safety concerns (e.g., sweet potatoes due to the toxicity of the leaves and stems, and cherry tomatoes due to the choking hazard for children). To establish the gardens, we held a one-hour early spring kick-off event at each intervention center, and invited parents and other family members, providers, and children to help plant the gardens.

The Watch Me Grow curriculum was introduced by the research team at the center kick-off event and was reinforced monthly during regular technical assistance visits by the health educator. The curriculum included an overview module, followed by monthly modules designed around a specific crop. Each month, the health educator described four discrete activities included in each module so that center staff could deliver the activities to the children approximately weekly. At each technical assistance visit, the health educator communicated with center staff to ensure that delivery of the previous month’s module went as planned. The curriculum, which included an existing published children’s book for each target crop, encouraged connection with the crop of the month through each of four kinds of activities (Table 1). The curriculum and visits by the health educator encouraged center staff to act as positive role models in the garden and in the classroom, by giving them language to use when approaching novel situations and foods. For example, providers were encouraged to taste the garden produce during taste tests and to say positive things about the fruit or vegetable.

**Table 1:** Monthly garden and classroom activities for each target crop for the Watch Me Grow intervention

**Crop**	**Activity**	**Description**	**Garden**	**Classroom**
**Lettuce**	Reading	*The Secret Life of Mitch Spinach* by Feerick & Hillenbrand		•
	Garden	Spinach spies: chart planting, nurturing, growth and harvest	•	
	Classroom	Seed sorting: identifying lettuce and spinach seeds		•
	Taste Testing	Spinach stackers taste test		•
**Strawberries**	Reading	*Monsters Don’t Eat Broccoli* by Hicks		•
	Garden	What part do we eat? Discover edible parts of many crops	•	
	Classroom	Broccoli friends: creating broccoli friend from various shapes		•
	Taste Testing	Broccoli mops and stalk sticks with healthy dipping sauces		•
**Spinach**	Reading	*Do Lions Like Lettuce?* by Butterfield		•
	Garden	Lettuce sprouts: graphing lettuces by color and size	•	
	Classroom	Cut and color a paper salad		•
	Taste Testing	“Lettuce do a taste testing” of various types of lettuce		•
**Broccoli**	Reading	*First Strawberries* by Bruchac		•
	Garden	Where’s my strawberry? Discover stages of strawberry growth	•	
	Classroom	“I’m a Strawberry” song to learn about plants and harvest		•
	Taste Testing	Fun with strawberry smoothies		•

To help ensure garden success, we provided gardening expertise through regular (i.e., at least monthly) visits by a study gardener. We also provided each intervention center with a digital camera, allowing the center director or teacher to photograph the garden and email pictures of plants, soil, or other conditions that appeared concerning. This method of troubleshooting helped prevent failure from disease or insufficient maintenance. We also provided small, inexpensive terra cotta “worms” that remained in the garden and alerted providers that plants were in need of water.

To reinforce integration of garden produce into the child-care menu, a health educator met with the provider at the center who was directly responsible for food purchasing and menu planning. These visits occurred every month of the intervention to help centers explore low-cost, sustainable ways to increase the number and nutritional quality of vegetables and fruits provided to children in care, beyond what the garden would produce.

### Outcome measure

To assess the main outcome for this study, we employed a structured dietary observation method developed by Ball *et al*[24] for use with preschool-aged children attending child care. Per protocol described in Ball *et al*[24], we randomly selected a classroom and then three children within that classroom for dietary observation at intervention and control centers before and after the intervention. Therefore, the same three children may not have been observed pre- to post-intervention. A trained Registered Dietitian, blinded to treatment group, conducted the dietary assessments. We observed all meals and snacks provided to children by the center over two full days of care. Amounts of all foods and beverage served, consumed, and wasted (i.e., spilled, traded, or discarded) were recorded for each of the three target children.

To assess the nutritional value and food groupings of foods and beverages, we used Nutrition Data System for Research (NDS-R) software (version 2008, Nutrition Coordinating Center, University of Minnesota, Minneapolis, MN). We reviewed dietary data to determine the amount and type of foods and beverages served to children averaged over two full days of care, focusing on vegetables and fruits. We compared food group values to the USDA MyPlate recommendations by age for each food group. We examined total vegetables, and reported on dark vegetables and potatoes separately. Dark vegetables were counted both separately and as part of total vegetables given their high nutrient density. We did not include vegetable and fruit juices when computing total servings of vegetables and fruits.

### Other measures

We assessed demographic variables of centers, including years of operation, participation in the Child and Adult Care Food Program (CACFP), a federal food assistance program that provides reimbursement for eligible meals and snacks served to low-income children in child care, number of children enrolled, and number of state-subsidized children enrolled, meaning some children in care came from low-income families who received financial support from the state to pay for child care. Additionally, we collected data on the center director’s race/ethnicity and level of education.

### Analysis

We used the NDS-R Serving Count Food file, which contained the daily totals for food group serving counts based on servings defined by the Dietary Guidelines for Americans 2005. We computed difference scores and standard deviations (SD) of average daily vegetable and fruit servings served to and consumed by children pre- to post-intervention. Due to sample size limitations, we did not conduct formal statistical analysis beyond comparing crude differences in mean servings of vegetables and fruits. Calculations were conducted using SAS version 9.2 (SAS Institute, Cary, NC).

## Results

The four child-care centers in the sample were in operation an average (SD) of 7.5 (4.8) years and two (50%) participated in CACFP (1 intervention and 1 control). An average of 19.0 (7.9) children were enrolled per center, with over half (73%) being three, four, or five years of age—the other children in care were younger than three years. All centers had at least some subsidized children enrolled. All center directors were female, 75% were African American, and 50% had a college degree. Both intervention center gardens produced at least some of the four target crops.

When we examined mean (SD) servings of vegetables and fruits, we found that post intervention, children in the intervention centers were served slightly fewer (0.18 (0.63)) servings of vegetables, but consumed more of what they were served (0.25 (1.10)) than controls (Table 2). These additional vegetables included both dark vegetables and potatoes. Children in the control centers were served (−0.37 (0.36)) and consumed (−0.18 (0.52)) fewer vegetables pre- to post-intervention, with no change in dark vegetables served or consumed. Less fruit was served post-intervention to children in both intervention (−0.33 (0.72)) and control (−0.10 (0.52)) centers. However, children in control centers consumed more servings of fruit post-intervention (0.15 (0.25)) than children in the intervention centers (−0.33 (0.72)).

**Table 2:** Servings and consumption of vegetables and fruits in the watch me grow intervention*

	**Intervention**						**Control**					
	**Served**			**Consumed**			**Served**			**Consumed**		
	***Mean (SD)***						***Mean (SD)***					
	**Pre**	**Post**	**Diff**	**Pre**	**Post**	**Diff**	**Pre**	**Post**	**Diff**	**Pre**	**Post**	**Diff**
**Vegetables**	1.42 (0.67)	1.24 (0.57)	−0.18 (0.63)	0.80 (0.68)	1.05 (0.67)	0.25 (1.10)	1.13 (0.31)	0.75 (0.21)	−0.37 (0.36)	0.80 (0.38)	0.63 (0.28)	−0.18 (0.52)
Dark	0.19 (0.34)	0.07 (0.14)	−0.11 (0.40)	0.00 (0.00)	0.07 (0.13)	0.07 (0.13)	0.01 (0.02)	0.02 (0.04)	0.00 (0.01)	0.01 (0.02)	0.02 (0.01)	0.01 (0.05)
White potatoes	0.17 (0.23)	0.35 (0.37)	0.18 (0.57)	0.17 (0.23)	0.33 (0.34)	0.16 (0.54)	0.22 (0.39)	0.00 (0.00)	0.22 (0.39)	0.22 (0.39)	0.00 (0.00)	0.22 (0.39)
**Fruit**	1.55 (0.99)	0.92 (0.56)	−0.62 (0.58)	1.00 (0.89)	0.67 (0.22)	−0.32 (0.72)	0.59 (0.27)	0.49 (0.40)	−0.10 (0.52)	0.32 (0.29)	0.46 (0.43)	0.15 (0.26)

*Based on USDA MyPlate serving sizes by age group for each food group (vegetables, fruits).

## Discussion and conclusion

We found that children in the intervention centers consumed, on average, an additional 1/4 serving of vegetables, while children in control centers decreased their vegetable intake by 1/5 of a serving. Children in intervention centers consumed greater quantities of vegetables, despite the fact that their centers served slightly fewer vegetables from baseline to follow up. The intervention may have had less of an effect on center staff behavior (i.e., centers were not necessarily more likely to put more vegetables on the table), but it did appear to affect children’s acceptance and consumption of vegetables, in that they were more likely to eat vegetables that were put on their plates. The relative increase in consumption of vegetables by children in intervention centers suggests that there may have been increased acceptance of vegetables by children exposed to the Watch Me Grow intervention. However, the sample size limits our ability to conduct statistical analyses.

We did not observe an increase in the amount of fruit served to or consumed by children. On the contrary, we found a decrease in fruit servings in our intervention centers. This may be due to the fact that the intervention included three vegetables (lettuce, broccoli, and spinach) and only one fruit (strawberries). Including additional fruits as target crops may have changed our results. But, children consumed a variety of vegetables at each dietary observation period (before and after the intervention), not just the target intervention vegetables.

Our findings of increases in vegetables only, while crude, are consistent with those found in garden-based intervention studies in older children [25], and these types of studies are becoming more common. In recent years, interest in school-based gardening interventions has gained momentum with French *et al*[26]. promoting school gardens as a unique approach to encourage vegetable and fruit intake in children. Thus far, the majority of interventions have focused on nutrition curricula that do not include actual fruit and vegetable gardens, with varying results [27-30]. Interventions that encourage hands-on experiences and include home-grown produce have shown to increase consumption beyond that of an intervention that merely increases fruit and vegetable availability [19,31]. Despite this potential benefit, current research on gardening interventions shows mixed results [7,28]. While some studies have not shown an increase in fruit or vegetable consumption, they have seen positive results in vegetable and fruit identification [27,29], greater willingness to taste fruits and vegetables [27], and an increased preference for certain fruits and vegetables [25,30]. A few studies, however, have seen positive changes in fruit and vegetable consumption. McAleese and colleagues [25] compared a sixth grade vegetable and fruit intervention with garden components against that same intervention without garden components and a control, and found that vegetable and fruit consumption was higher in the garden group [32]. Wang *et al*[33] found that increased exposure to a school-based garden intervention yielded an increase in one half a serving of vegetables from baseline to follow up. However, previous studies have not focused on younger children, and none have targeted child care for intervention.

### Implications for research and practice

The Watch Me Grow pilot intervention showed promise, but gardening interventions require a substantial number of resources (e.g., time and funding to establish the gardens, center buy-in, and parent support). Thus, for this feasibility study we restricted our sample size to a small number of centers, which also limited our ability to conduct formal statistical testing to evaluate intervention effects. Additional research should increase the number of child-care centers for a larger evaluation trial, allowing for sufficient testing of the null hypothesis. We also did not measure children’s familiarity with our target vegetables and fruits, or their willingness to taste them prior to the intervention. Moreover, we were not able to separate the intervention components (i.e., garden, classroom curriculum, technical assistance from a health educator) to assess individual impact on child vegetable consumption. We were not able to determine which activities may have had the most impact; center staff reported that the activities were typically well-received by children, but some were more appropriate for younger children (e.g., songs) and some for older children (e.g., seed sorting activities). Staff reported that the monthly modules engaged all children in at least one activity. The primary limitation of this study, however, was the small sample size, which restricted our ability to formally evaluate the program with appropriate statistical testing.

The pilot was also geographically limited to Zone 7 and was conducted in the spring and early summer months—the ideal seasons for growing produce. This intervention may not work as well in colder climates or in other seasons. Additionally, there were some outdoor space requirements for this intervention and centers in more urban settings may not be able to grow sufficient quantities of vegetables and fruits to impact child intake. Further evaluation studies, powered to detect differences by group, are needed to better understand how garden-based interventions can impact vegetable and fruit consumption in child care. These studies should also consider including a strong staff component to better affect what is being served, and a parent component to reach beyond the child-care setting to impact vegetable and fruit intake in the family home. Despite these limitations, the Watch Me Grow pilot intervention showed potential to increase vegetable consumption in preschoolers.

## Competing interests

The authors declare that they have no competing interests.

## Authors’ contributions

SEBN conceived of the study, and participated in its design and intervention development. RJNB participated in the design of the study, led the development of the intervention, and oversaw its implementation. Both authors read and approved the final manuscript.

## Pre-publication history

The pre-publication history for this paper can be accessed here:

http://www.biomedcentral.com/1471-2458/13/363/prepub
